# Semaglutide-Induced Lupus Erythematosus With Multiorgan Involvement

**DOI:** 10.7759/cureus.55324

**Published:** 2024-03-01

**Authors:** Vanessa Castellanos, Hiwot Workneh, Ayesha Malik, Bijal Mehta

**Affiliations:** 1 Internal Medicine, Hackensack Meridian Mountainside Medical Center, Montclair, USA; 2 Internal Medicine, Hackensack Meridian Mountainside Medical, Montclair, USA

**Keywords:** drug induced hepatitis, autoimmune hepatitis, semaglutide, drug-induced cutaneous vasculitis, glp-1 agonist, drug-induced lupus (dil)

## Abstract

We report the case of a 76-year-old female who presented with a new onset of petechial rash in her lower extremities after the introduction of a new agent, semaglutide. She started taking this medication three months before her presentation at an initial dosage of 0.5 mg subcutaneously every week. She noticed a 15-pound weight loss and debilitating fatigue within that timeframe. She stopped taking the medication due to nontolerance and GI upset (nausea and vomiting) about a week before her hospitalization. She denied the use of any other agents.

Initial lab work revealed elevated transaminases, alkaline phosphatase, total bilirubin, and inflammatory markers. A CT of the abdomen revealed mild cirrhosis and hepatosplenomegaly. Other causes for cirrhosis were effectively ruled out with negative viral hepatitis, ceruloplasmin levels, and the HFE gene. An autoimmune panel was conducted, yielding positive antinuclear antibody (ANA), anti-histone antibodies, elevated double-stranded DNA, as well as low complement levels supporting evidence of drug-induced lupus (DIL). Anti-mitochondrial M2 and anti-smooth antibodies were also detected, indicating a possible overlap syndrome with autoimmune hepatitis. Perinuclear anti-neutrophil cytoplasmic antibodies (P-ANCA) and anti-neutrophil cytoplasmic autoantibodies (C-ANCA) were negative and ruled out the possibility of ANCA-associated vasculitis.

The patient's condition improved with pulse-dose steroids, leading to an improvement in liver function tests. Consequently, the decision to perform skin and liver biopsies was deferred. She was discharged with a tapering dose of steroids and scheduled for outpatient follow-up to monitor her progress. This case report can offer insights to healthcare providers regarding the potential side effects of GLP-1 RAs in their patient population.

## Introduction

Drug-induced lupus (DIL) erythematosus is an autoimmune phenomenon resulting from exposure to certain drugs with clinical manifestations similar to systemic lupus erythematosus (SLE) after exposure to certain drugs [1,2]. We present the case of a 76-year-old female who exhibited clinical and laboratory evidence of autoimmune reactions following the recent use of semaglutide, a GLP-1 receptor agonist. Her symptoms, positive autoantibodies, and liver abnormalities suggested a potential DIL with a vasculitic component and a possible overlap with autoimmune hepatitis.

## Case presentation

A 76-year-old female with a past medical history of hypertension, hypothyroidism, intermittent asthma, and pre-diabetes presented with a three-week course of new-onset petechial rash in her lower extremities. She reported the recent use of semaglutide (Wegovy) 0.5 mg subcutaneously every week three months ago, which she discontinued a few days before her hospitalization due to GI upset (nausea and vomiting). There were no other new medications. Her current medications were amlodipine 10 mg, bupropion XL 300 mg, levothyroxine 75 mcg, fluticasone propionate-salmeterol (Advair) inhaler, montelukast 10 mg, atorvastatin 20 mg, losartan 100 mg, and omeprazole 10 mg. In a review of systems, she had lost about 15 lbs in three months and reported debilitating fatigue with non-restorative sleep. She denied muscle weakness, joint involvement, alopecia, and numbness or tingling in the upper or lower extremities. On a physical exam, a diffuse petechial rash was noted in the lower extremities, which spread to her lower abdomen. The rash was non-pruritic, non-blanchable, non-raised, and painless. There was no evidence of skin or mouth ulcers, and palpable hepatomegaly was noted. She denied any relevant family history, previous history of liver disease, recent travel, or social history.

**Figure 1:**
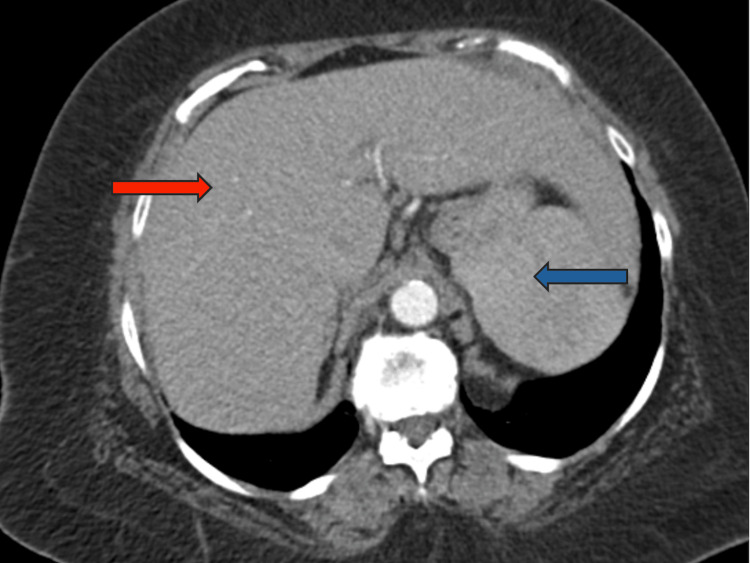
Axial CT scan of the abdomen showing mild cirrhosis (red arrow) and splenomegaly (blue arrow)

Initial laboratory examination revealed elevated alanine transaminase (ALT) of 306 U/L, aspartate aminotransferase (AST) of 431 U/L, alkaline phosphatase (ALP) of 219 U/L, c-reactive protein (CRP) of 42 mg/L, erythrocyte sedimentation rate >130 mm/hr, total bilirubin of 5.6 mg/dl, direct bilirubin of 4.2 mg/dl, and low hemoglobin of 10 g/dL. The protein/creatinine ratio was normal. Further investigations were conducted to exclude other potential causes of cirrhosis. Serological tests for viral hepatitis (hepatitis A virus (HAV) antibody), qualitative hepatitis B surface antibody (HBsAb), hepatitis B surface antigen (HBsAg), total hepatitis B core antibody (HBcAb), HCV antibody, ceruloplasmin level, and the HFE gene associated with hemochromatosis yielded negative results. A CT scan of the abdomen and pelvis, conducted without contrast (Figure 1), indicated mild cirrhosis and splenomegaly. The magnetic resonance cholangiopancreatography (MRCP) did not reveal any significant findings, except for the presence of hepatosplenomegaly; however, this was limited due to significant motion artifacts. 

Based on her presentation with a vasculitic suspicious rash and fatigue, an autoimmune panel was conducted, yielding the following results: positive ANA direct, elevated double-stranded DNA at 123 IU/ml, positive anti-histone antibody, low C4 (5 mg/dl), and normal C3 (92 mg/dl) levels. Additionally, the presence of anti-mitochondrial M2 and anti-smooth antibodies was detected. However, anti-Sjögren's syndrome-related antigen A (SSA) (Ro), anti-SSB (La), perinuclear anti-neutrophil cytoplasmic antibodies (P-ANCA), and anti-neutrophil cytoplasmic autoantibody (C-ANCA) were all negative.

Given the patient's history of exposure to a new agent with the presence of ANA, anti-histone antibodies, elevated dsDNA, and low C4, she met the American College of Rheumatology (ACR)/European League Against Rheumatism (EULAR) criteria for diagnosis of SLE, likely secondary to the drug agent. Furthermore, liver involvement with elevated liver function testing is most consistent with active systemic inflammatory processes like SLE or vasculitis; however, ANCA-associated vasculitis is less likely in the absence of P-ANCA and C-ANCA. During the hospital stay, the patient was administered high doses of steroids (1 g of methylprednisone daily for three days), which led to an improvement in liver function tests (LFTs). In light of this positive response to treatment, the decision to perform a skin and liver biopsy was deferred. She was discharged with a tapering dose of steroids and scheduled for outpatient follow-up.

## Discussion

Drug-induced lupus can be caused by various medications, and the list continues to expand as new biologic agents are introduced into medical practice [1]. It typically presents with mild lupus-like symptoms, including joint pain, muscle pain, fever, and skin manifestations [1]. During the patient’s initial assessment, her medications were reviewed, and none were found to be associated with DIL [3,4]. Considering the patient's clinical presentation and recent initiation of a new medication, the initial clinical diagnosis included two potential conditions based on similar previously reported cases: GLP-1 agonist-associated vasculitis and autoimmune hepatitis [5].

Drug-induced small vessel vasculitis manifests as inflammation in the small blood vessels, predominantly affecting the skin and presenting with symptoms like palpable purpura, petechiae, or skin ulcers. Various medications have been linked to this condition, and its diagnosis involves evaluating the patient's medical history, medication usage, and clinical symptoms, and performing a skin biopsy for confirmation [6,7].

As part of a workup to rule out systemic involvement, the patient's auto-immune panel revealed positive ANA direct, dsDNA, and low C4 levels. Anti-mitochondrial M2 and anti-smooth antibodies were also found to be positive [8]. After discontinuing the presumed offending agent (semaglutide) and initiating high-dose steroids, the patient's symptoms were notably improved, which further supports the diagnosis of DIL.

The GLP-1 receptor agonists, commonly referred to as incretin mimetics, are medications that are increasingly being utilized due to their proven efficacy in the treatment of type 2 diabetes mellitus and obesity [9]. Semaglutide is an analog of native GLP-1 RA with slight modifications, has recently gained approval, and is renowned for its effectiveness and favorable safety profile [10]. Given its recent introduction to the market, specific reports of autoimmune reactions and related side effects have not been extensively studied yet [11,12]. However, in recent post-marketing surveillance based on reports from individuals who experienced side effects while using Ozempic, SLE-like symptoms were reported by seven out of 12,332 individuals (0.06%). This adverse effect has been predominantly reported in females over the age of 60, particularly among those who have been using the medication for less than one month [13]. Yet, a definitive correlation with this phenomenon has not been firmly established.

## Conclusions

Limited available data exists regarding the association between GLP-1 agents and the development of DIL, or autoimmune hepatitis. Our case can provide a valuable reference for future studies investigating such effects in patients with pre-existing autoimmune conditions or not. This case report can offer insights to healthcare providers regarding the potential side effects of GLP-1 RAs in their patient population.
